# Investigation of the Noise Emitted from Elevated Urban Rail Transit Paved with Various Resilient Tracks

**DOI:** 10.3390/ma18050968

**Published:** 2025-02-21

**Authors:** Quanmin Liu, Kui Gao, Yifei Miao, Lizhong Song, Si Yue

**Affiliations:** 1 Engineering Research Center of Railway Environmental Vibration and Noise of Ministry of Education, East China Jiaotong University, Nanchang 330013, China; qmlau007@126.com (Q.L.); gk13605524797@163.com (K.G.); hemiao006@126.com (Y.M.); 2 China Railway Siyuan Survey and Design Group Co., Ltd., Wuhan 430063, China; 006188@crfsdi.com

**Keywords:** urban rail transit, resilient track, wheel–rail noise, structure-borne noise, total noise

## Abstract

Based on the dynamic receptance method, a vehicle–track–bridge interaction model was developed to calculate the wheel–rail interaction forces and the forces transmitted to the bridge in an elevated urban rail transit system. A prediction model integrating the finite element method–boundary element method (FEM-BEM) and the statistical energy analysis (SEA) method was established to obtain the noise from the main girder, track slab, and wheel–rail system for elevated urban rail transit. The calculated results agree well with the measured data. Thereafter, the noise radiation characteristics of a single source and the total noise of elevated urban rail transit systems with resilient fasteners, trapezoidal sleepers, and steel spring floating slabs were investigated. The results demonstrate that the noise prediction model for elevated urban rail transit that was developed in this study is effective. The diversity of track forms altered the noise radiation field of elevated urban rail transit systems significantly. Compared to monolithic track beds, where the fastener stiffness is assumed to be 60 × 10^6^ N/m (MTB_60), steel spring floating slab tracks (FSTs), trapezoidal sleeper tracks (TSTs), and resilient fasteners with a stiffness of 40 × 10^6^ N/m (MTB_40) and 20 × 10^6^ N/m (MTB_20) can reduce bridge-borne noise by 24.6 dB, 8.8 dB, 2.1 dB, and 4.2 dB, respectively. These vibration-mitigating tracks can decrease the radiated noise from the track slab by −0.7 dB, −0.6 dB, 2.5 dB, and 2.6 dB, but increase wheel–rail noise by 0.4 dB, 0.8 dB, 1.3 dB, and 2.4 dB, respectively. The noise emanating from the main girder and the track slab was dominant in the linear weighting of the total noise of the elevated section with MTBs. For the TST and FST, the radiated noise from the track slab contributed most to the total noise.

## 1. Introduction

To alleviate the escalating traffic congestion in major cities in China, urban rail transit systems have undergone rapid expansion, emerging as an increasingly dense operational network. Compared to underground lines, elevated lines offer several advantages, including a lower infrastructure cost, a shorter construction period, and easier maintenance access. They constitute a vital component of urban rail transit systems. By the end of 2024, the total operational length of elevated lines in China exceeded 2000 km. However, the noise generated by these elevated urban rail transit systems has caused considerable disturbance to residents living near the lines [1,2].

The wheel–rail interaction generates huge shock forces due to rail irregularities. The rails on bridges often exhibit quite light damping, resulting in quite large vibrations and noise. The bridge, subject to the dynamic force transmitted from the wheel–rail interaction, will emit structure-borne noise. The speed of trains running on urban rail transit lines is usually lower than 100 km/h, so the aerodynamic noise of the train is negligible. Therefore, the main sources of noise in elevated urban rail transit include the rolling noise and bridge structure-borne noise [3].

During the operation of the train, the track structure plays a crucial role in transmitting vibration energy and supporting the train load, ultimately transferring it to underlying foundation structures, such as bridges. The resilient track is a critical measure in mitigating the vibration along rail transit lines. Different track configurations inevitably influence the amplitude and distribution of wheel–rail noise, as well as bridge structure-borne noise. In other words, the use of resilient tracks alters the wheel–rail interaction dynamics, modifying the excitation experienced by the tracks. This, in turn, affects the track slab noise and wheel–rail noise, as well as their contributions to the overall sound pressure levels. Therefore, determining the sound field distribution of elevated urban rail transit lines paved with various typical track configurations holds significant importance in the assessment of noise impact.

The vibration mitigation measures commonly used in track design include elastic fasteners [4,5,6,7], trapezoidal sleepers [6,8], rubber floating slabs [9,10,11], steel spring floating slabs [8,12,13,14], and composite rubber–steel spring floating slab tracks [15] to reduce the vibration energy transmitted from trains to bridges. However, the vibration and radiated noise of the rail will be amplified when high-elasticity fasteners and trapezoidal sleepers are employed to reduce the bridge’s vibration and structure-borne noise [6]. Compared with embedded sleepers, lines equipped with steel spring floating slabs and rubber pad floating slabs exhibit significantly higher peak values of wheel–rail interaction forces [8,10], which intensify wheel–rail interactions and, consequently, increase radiated noise. In other words, reducing the stiffness of the track structure to diminish bridge structure-borne noise might inadvertently elevate wheel–rail noise. Therefore, further research on wheel–rail noise in resilient tracks is essential to explore the distribution characteristics of elevated urban rail transit noise under these conditions.

Extensive testing [1,3,12,16,17,18] has demonstrated that the frequency distribution of elevated urban rail transit noise predominantly falls within the range of 20 to 5000 Hz. Specifically, wheel–rail noise predominantly occurs within the medium- to high-frequency band of 500–2000 Hz [19], while bridge structure-borne noise is mainly concentrated in the low-frequency band below 200 Hz [20,21,22].

The prediction models of wheel–rail noise primarily encompass analytical and numerical models. Thompson [23] regarded the wheel as a finite element model and used the Timoshenko beam model to simulate the rail, replacing the Euler beam model in the Remington model, fully considering the high-frequency vibration of the rail. On this basis, the wheel–rail noise prediction software TWINS [24] was developed. Zhang et al. [25,26,27,28,29] further enhanced the TWINS model by incorporating factors such as ground reflection, acoustic radiation from sleepers and track slabs, sound absorption by the ballast, and the effects of rail shields. The numerical model’s calculation methods [30,31,32] mainly consist of the finite element method (FEM) and boundary element method (BEM). He et al. [31] employed FEM and BEM to calculate wheel and track noise, analyzing the impact of rubber layers on wheel–rail noise.

The prediction of bridge structure-borne noise typically relies on numerical methods, primarily the FEM-BEM and statistical energy analysis (SEA). Song et al. [32] predicted bridge structure-borne noise using a combination of the FEM and BEM. Although the BEM [22,33] is extensively utilized for structure-borne acoustics calculations, its efficiency markedly decreases when addressing noise up to 5000 Hz in urban rail transit systems. Therefore, the BEM is more suitable for predicting bridge structure-borne noise at frequencies below 200 Hz [19,20,23,34]. In contrast, SEA [35,36,37,38] assumes that energy is averaged over modes in medium- to high-frequency ranges, ensuring the validity and accuracy of the calculation results within these bands. However, its accuracy is relatively limited in low-frequency bands.

Therefore, in this study, the BEM was employed to calculate low-frequency noise, while the SEA was utilized to assess the medium- to high-frequency noise of elevated urban rail transit systems with resilient fasteners, monolithic track beds (MTBs), trapezoidal sleeper tracks (TSTs), and steel spring floating slab tracks (FSTs), respectively. The influence of track forms on the total noise of elevated urban rail transit systems was analyzed. Firstly, the analytical vehicle–track–bridge-coupled vibration model for the aforementioned three types of tracks was established using the dynamic receptance method [8]. The model was used to calculate the wheel–rail forces and the forces transferred from the track to the bridge. Secondly, the finite element method (FEM) was used to construct the bridge model, and a harmonic response analysis of the bridge excited by the force transferred from the track was carried out to obtain the vibration response of the bridge. The bridge structure-borne noise was then calculated using the BEM, where the bridge vibration from the harmonic response analysis was used as an input. Thirdly, finite element models of the wheel and rail–track system were established, and the wheel–rail forces were applied to obtain the low-frequency vibration responses of the wheel and rail–track system. The low-frequency acoustic radiation from the wheel and rail–track structure were subsequently calculated using the BEM. Fourthly, a wheel–rail noise prediction model based on SEA was developed, using the wheel–rail force as the input excitation to predict the wheel–rail noise in the medium- and high-frequency bands. Finally, based on the aforementioned calculation results, the distribution patterns of the bridge structure-borne noise, track slab noise radiation, wheel–rail noise, and total noise under resilient tracks were investigated.

## 2. Methodology for the Noise Prediction of Elevated Urban Rail Transit

**Figure 1:**
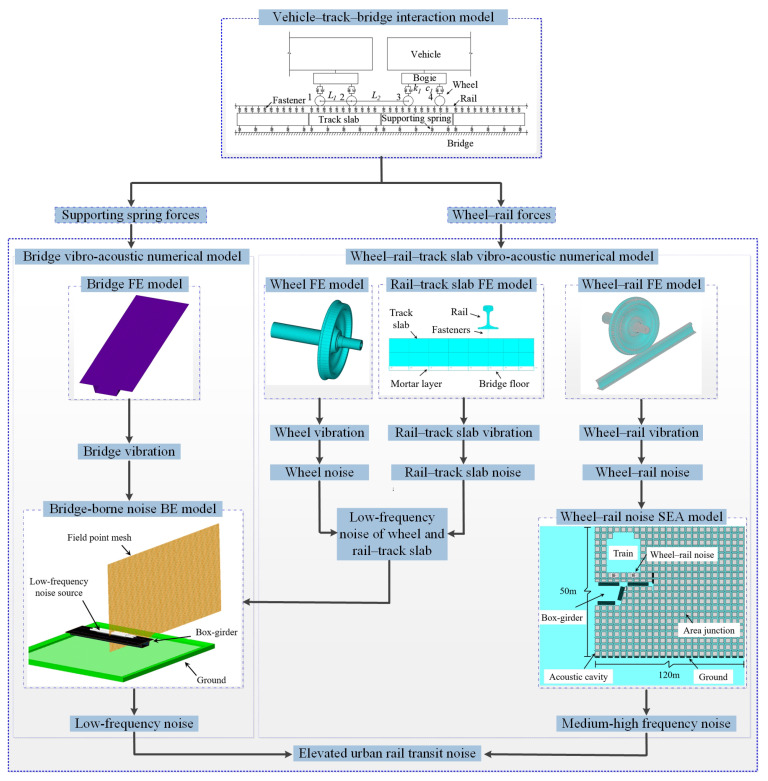
Noise prediction model for elevated urban rail transit systems.

The noise prediction model for elevated urban rail transit comprises four sub-models, as illustrated in Figure 1: (1) the vehicle–track–bridge interaction model; (2) the vibro-acoustic numerical model to simulate bridge structure-borne noise; (3) the low-frequency numerical model for wheel and rail–track slab noise; and (4) the medium- to high-frequency numerical model for wheel–rail noise. The vehicle–track–bridge interaction model is utilized to compute both the wheel–rail interaction force and the force transmitted to the bridge (supporting spring force). These forces serve as input excitations applied to the vibro-acoustic numerical models of the wheel–rail–track slab and the bridge, respectively. Harmonic response analysis was conducted on the finite element models of the wheel–rail–track slab system and bridge to derive their vibration responses. Subsequently, using these boundary conditions, the radiated noise from each sub-model was calculated. Specifically, the low-frequency noise of the wheel and rail–track slab was incorporated into the bridge structure-borne noise BE model as a line sound source to jointly determine the low-frequency sound field. Meanwhile, the medium- and high- frequency noise of the wheel–rail was applied as acoustic excitation in the SEA model to calculate the diffuse sound field. Finally, the two sound fields were superimposed to obtain the noise distribution of the elevated urban rail transit system.

### 2.1. Vehicle–Track–Bridge Interaction Model

The vehicle–track–bridge interaction model was developed and implemented using MATLAB, whose version number is 9.4.0.813654 (R2018a). In the vehicle–track–bridge interaction model, only the primary suspension is taken into account, since the natural frequency of the vehicle’s secondary suspension is approximately 1 Hz [8], which is lower than the analysis frequency for viaduct noise. The wheel–rail contact is modeled as a linear Hertzian contact [19]. The rail is represented as an infinitely long Timoshenko beam discretely supported by fasteners. The fasteners and supports beneath the track slab are discretized as linear springs characterized by specified stiffness and damping coefficients. The track slab is modeled as a finite-length Euler–Bernoulli beam [16]. The simply supported bridge is used to carry the track, and its vertical stiffness is much higher than that of the track, so the bridge is modeled as a rigid body herein [37]. In Figure 1, *L*_1_ denotes the wheelbase, while *L*_2_ indicates the distance between the nearest bogies of the vehicle. The numbers 1, 2, 3, and 4 represent the four wheels in the model. Generally, the maximum operating speed of urban rail transit vehicles ranges from 80 to 160 km/h, which is significantly lower than the propagation speed of vibration waves in the rail. Consequently, the moving irregularity model [8] can be employed to calculate the wheel–rail interaction force in the frequency domain. Thus, the wheel–rail force was calculated as follows:

(1) $$F=-\frac{R}{\alpha^{w}{+\alpha }^{r}+\left({1/K}_{H}\right)}$$

where, *α*^w^ denotes the dynamic receptance of the wheel; *α*^r^ represents the dynamic receptance at the wheel–rail contact point of the rail; *K*_H_ = 1/*α*^c^, where *α*^c^ is the linear dynamic receptance at the wheel–rail contact spring; and *R* signifies the wheel–rail roughness.

**Figure 2:**
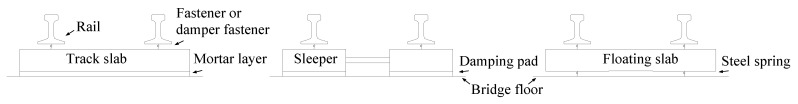
Three typical resilient track structures.

This study focuses on the radiation characteristics of noise from elevated urban rail transit systems with a box-girder bridge paved with several types of track. Specifically, the noise levels for tracks equipped with resilient fasteners, trapezoidal sleepers, and steel spring floating slabs are assessed. The cross-sectional schematic of the resilient track structure is illustrated in Figure 2.

Based on the characteristics of layered structures, the three types of resilient tracks illustrated in Figure 2 can be simplified as the track model depicted in Figure 1. Normal and resilient fasteners are modeled as linear springs with distinct stiffness and damping properties. The mortar layer beneath the monolithic track bed, the rubber pad under the trapezoidal sleeper, and the steel spring within the floating slab are also represented by linear springs with specific stiffnesses and damping values.

### 2.2. Prediction Model for the Structure-Borne Noise of the Box-Girder

The structure-borne noise of the box-girder bridge is calculated using the FEM-BEM, as illustrated in Figure 1. In the FEM modeling, the 30 m-span, simply supported box-girder is constructed based on its actual dimensions using shell elements, and the piers are simplified as the local constraint conditions at the bottom plate of the bridge ends. The forces transferred to the bridge, as determined by the vehicle–track–bridge interaction model, are applied to the FEM model for harmonic response analysis. Subsequently, the vibration displacements of the bridge serve as the input excitations of the BEM model to calculate the acoustic radiation.

### 2.3. Prediction Model for the Low-Frequency Noise of the Wheel–Rail–Track Slab

The interaction between the wheels of running trains and rails generates wheel–rail vibration noise radiation, which significantly influences the distribution of environmental noise around elevated urban rail transit systems [39]. Trapezoidal sleepers and steel spring floating slab tracks exhibit significant noise reduction effects on bridge structure-borne noise, owing to the elastic components beneath the sleepers and slabs [8]. However, the vibration and sound radiation from the track slabs might become predominant sources. Since acoustic radiation from track slab vibrations predominantly influences noise levels in the frequency range below 500 Hz [40,41], and the applicability of the FEM-BEM and SEA method is complementary, the prediction for the noise generated from the wheel, rail, and track slab is divided into two frequency ranges to enhance computational efficiency. The low-frequency noise prediction model for the wheel, rail, and track slab system was established based on the FEM-BEM herein, where the wheel–rail interaction force causes excitation. The upper bound frequency is 562 Hz to cover the 1/3 octave center frequency of 500 Hz. The low-frequency wheel–rail–track slab noise is then treated as a line sound source and integrated into the bridge-borne noise prediction model to evaluate the low-frequency noise of elevated urban rail transit systems, as illustrated in Figure 1. The size of the acoustic area is set to 120 m × 50 m to observe the noise diffusion of the bridge structure.

### 2.4. Prediction Model for the Medium–High Frequency Wheel–Rail Noise

The FEM-BEM is combined with the SEA method to calculate the distribution of medium- and high-frequency wheel–rail noise. Firstly, the interaction forces between the wheel and rail are applied to the FEM model of the wheel and rail. Subsequently, the BEM is utilized to compute the sound power of the wheel–rail noise, which is then treated as a sound source within a SEA model, accounting for interactions with the bridge, the ground, and other ancillary structures. The propagation characteristics of medium- and high-frequency wheel–rail noise in the air can be determined through this model.

## 3. Validation of the Noise Prediction Model

### 3.1. Measurement Overview

**Figure 3:**
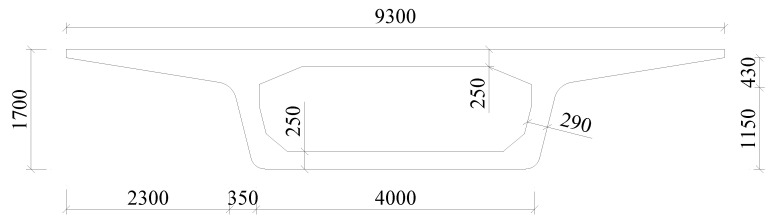
Cross-sectional diagram of the box-girder (unit: mm).

In the measurement, the vibration and noise data generated by a 30 m, simply supported, single-cell box-girder in an urban rail transit line were collected. The height of the double-track box-girder with MTBs is 1.7 m, the width of the top plate is 9.3 m, and the width of the bottom plate is 4 m. The distance from the bottom of the box-girder to the ground is approximately 4 m. The cross-sectional dimensions at mid-span are illustrated in Figure 3. The test vehicle was a four-car, L-type metro train and the running train speed was approximately 75 km/h.

The testing instruments included a 24-channel data acquisition device, PCB393B04 vertical vibration acceleration sensors, and free-field microphones. The sampling frequencies for the vibration acceleration and sound pressure are 1000 Hz and 25,000 Hz, respectively.

**Figure 4:**
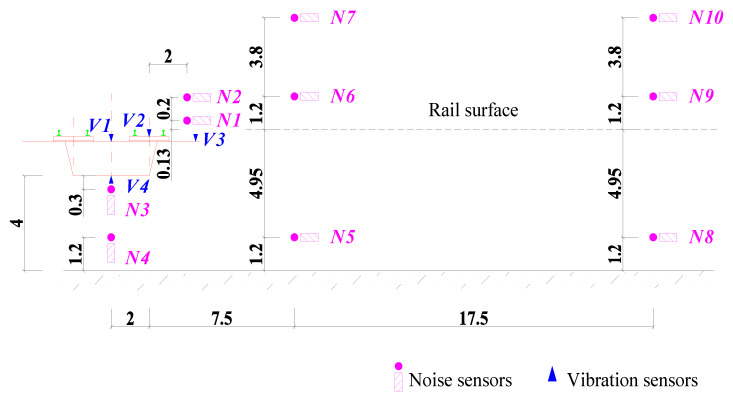
Layout of the noise and vibration measuring points (unit: m).

**Figure 5:**
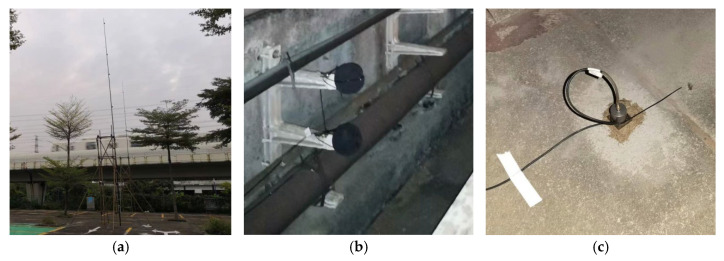
Photos of the test: (**a**) train and elevated bridges; (**b**) sound sensors; (**c**) vibration sensor.

Four accelerometers (V1–V4) and ten microphones (N1–N10) were placed at the mid-span section of the box-girder. The vibration measurement points V1–V4 were, respectively, used to obtain the vertical vibration acceleration levels of the top slab, the track slab, the flange slab, and the bottom slab of the box-girder. Noise sensors N1–N2 and N3–N4 were used to capture the trackside noise and structure-borne noise of the box-girder, while N5–N7 and N8–N10 were used to measure the sound pressure levels of the wayside noise at distances of 7.5 m and 25 m from the centerline of the track, respectively. The layout of the noise and vibration measurement points is shown in Figure 4, and the test photos are shown in Figure 5.

### 3.2. Verification of the Noise Prediction Model

**Figure 6:**
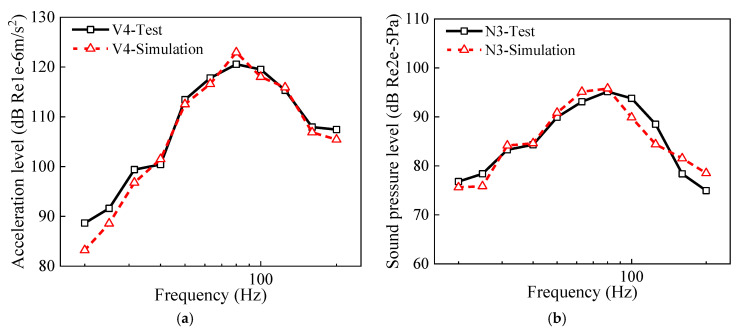
Measured and numerical results of the bottom plate for the bridge: (**a**) acceleration level; (**b**) sound pressure level.

The input rail roughness in the vehicle–track–bridge interaction model was derived from the ISO 3095-2013 [42]. The train speed in the calculation was consistent with that of the measurement. Other calculation parameters are detailed in Table 1. Owing to the shielding effect of the girder on the wheel–rail noise, the sound pressure measured beneath the bottom plate of the box-girder was predominantly attributed to the structure-borne noise of the girder. However, it is known that the sound pressure at the measurement points located farther from the box-girder includes both the wheel–rail noise and the bridge structure-borne noise. The calculated and measured values for the vibration and noise of the box-girder’s bottom plate are presented in Figure 6. It was found that the measured and calculated results for the vibration of the box-girder bottom plate at V4 are in good agreement, as are the measured and calculated sound pressure levels at noise measurement point N3. Therefore, it can be concluded that the bridge structure-borne noise prediction model is reliable.

**Figure 7:**
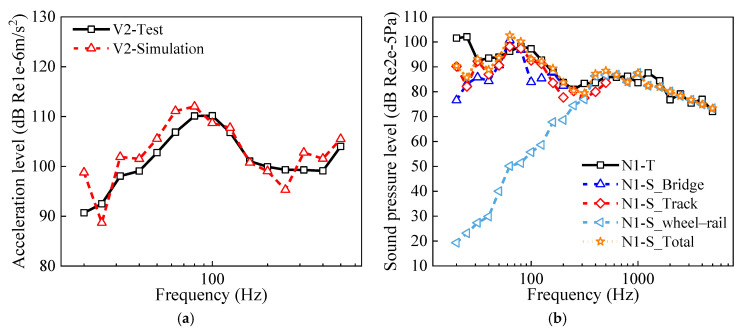
Measured and numerical results: (**a**) track slab vibration; (**b**) sound pressure level at N1.

The wheel–rail interaction force was applied to the FEM models of the wheel and the rail–track slab, and the wheel noise, as well as the radiated noise from the rail–track slab, was calculated using the BEM in the low-frequency band. These calculation results are presented in Figure 7. It was found that the predicted vibrations for the track slab were in good agreement with the measured values, and the calculated sound pressure levels at measurement point N1 agreed well with the measured results, as well.

In conclusion, the noise prediction model for elevated urban rail transit systems was validated. Moreover, as illustrated in Figure 7b, it was found that the sound pressure level at measurement point N1 was dominated by the bridge structure-borne noise and track slab radiation noise in the frequency range below 315 Hz. However, the wheel–rail noise was the primary contributor to the sound pressure levels in the frequency range above 315 Hz.

**Figure 8:**
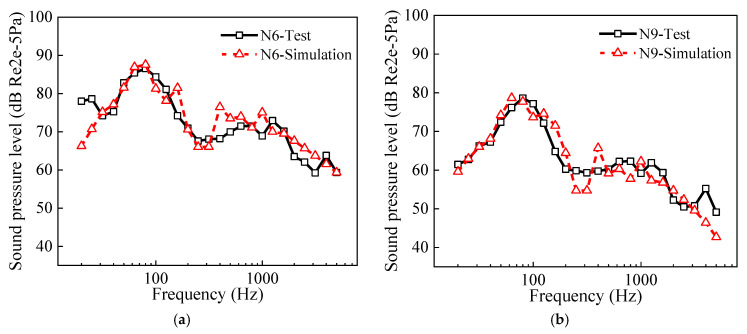
Measured and numerical noise around the elevated urban rail transit system: (**a**) N6; (**b**) N9.

The wheel–rail force was applied to the FEM model of the wheel and rail to compute their structural vibrations, which were then input into the BEM model to evaluate the sound power of the wheel–rail noise in the medium- to high-frequency range. Subsequently, a SEA model of the wheel–rail noise distribution was established to explore the propagation characteristics of the noise. Finally, by integrating the bridge structure-borne noise and the radiated noise from the wheel–rail–track slab system, the noise levels at points around the elevated urban rail transit were calculated. Figure 8 presents a comparative analysis of the measured versus calculated sound pressure levels at N6 and N9. It was found that the trends and magnitudes of the measured sound pressure levels agreed well with those of the numerical solutions, verifying the noise prediction model in this study again.

## 4. Comparison of Noise Levels of a Viaduct with Various Track Forms

To investigate the impact of vibration reduction measures on the noise of elevated urban rail transit, a MTB was used as a reference. The differences in bridge structure-borne noise, track slab radiation noise, and wheel–rail noise with the three types of tracks (resilient fasteners, trapezoidal sleepers, and steel spring floating slabs) are discussed. The calculation parameters in Table 1 are still adopted. Among them, the fastener stiffness of the conventional monolithic track bed was 60 × 10^6^ N/m (MTB_60), and the stiffness of the resilient fasteners was 40 × 10^6^ N/m (MTB_40) and 20 × 10^6^ N/m (MTB_20), respectively. The fastener stiffness of the TST and FST was the same as that of MTB_60. In addition, the distinctions between the FST, TST, MTB_20, MTB_40, and MTB_60 primarily resided in the variations in track slab dimensions and the stiffness of the spring support beneath the track slab, as listed in Table 1. Other calculation parameters remained the same.

### 4.1. Variations in Forces

**Figure 9:**
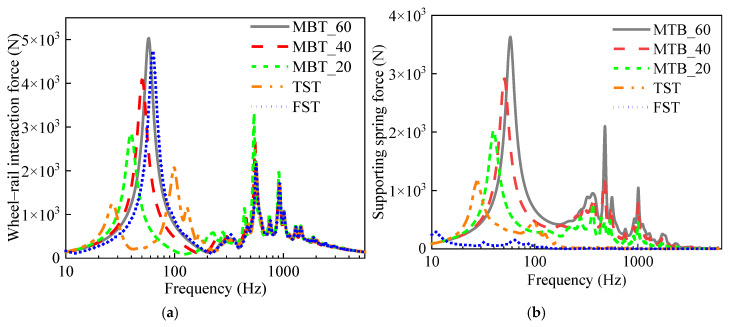
The force for different tracks: (**a**) wheel–rail force; (**b**) supporting spring force.

The wheel–rail interaction force and the supporting spring force under the slab derived from the vehicle–track–bridge interaction model are illustrated in Figure 9. In the low-frequency range, the peak value of the wheel–rail force for MTB_60 is slightly higher than that of the FST, and significantly higher than that of the TST. As the stiffness of the fasteners decreased, the peak value of the wheel–rail force for the MTB gradually diminished, and the peak frequency shifted toward lower frequencies. This indicates that low-stiffness fasteners may amplify low-frequency vibrations. In the frequency range above 315 Hz, the wheel–rail forces for the MTB, TST, and FST exhibited minimal differences. However, as the stiffness of the fasteners decreased, the peak value of the wheel–rail force for the MTB increased in this frequency range. The diminished dynamic receptance led to an increase in the wheel–rail force in this frequency range.

In the low-frequency band, the variation pattern of the supporting spring force aligned with that of the wheel–rail force. However, the peak value of the supporting spring force in the FST is the smallest among these tracks, indicating its effectiveness in significantly reducing the forces transmitted from the wheel–rail interface to the bridge structure. In the high-frequency band, the supporting spring forces, ranked from largest to smallest, are as follows: MTB_60, MTB_40, MTB_20, TST, and FST.

### 4.2. Variations in Sound Pressure Levels

**Figure 10:**
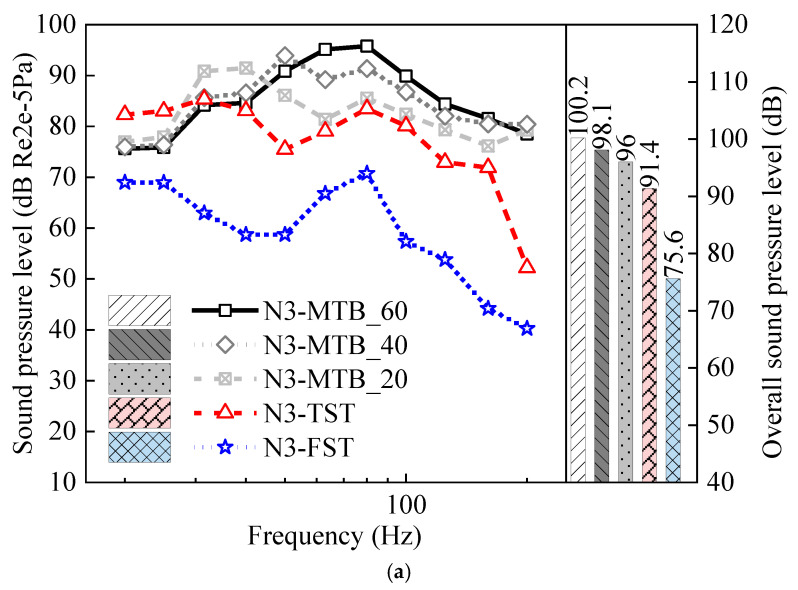

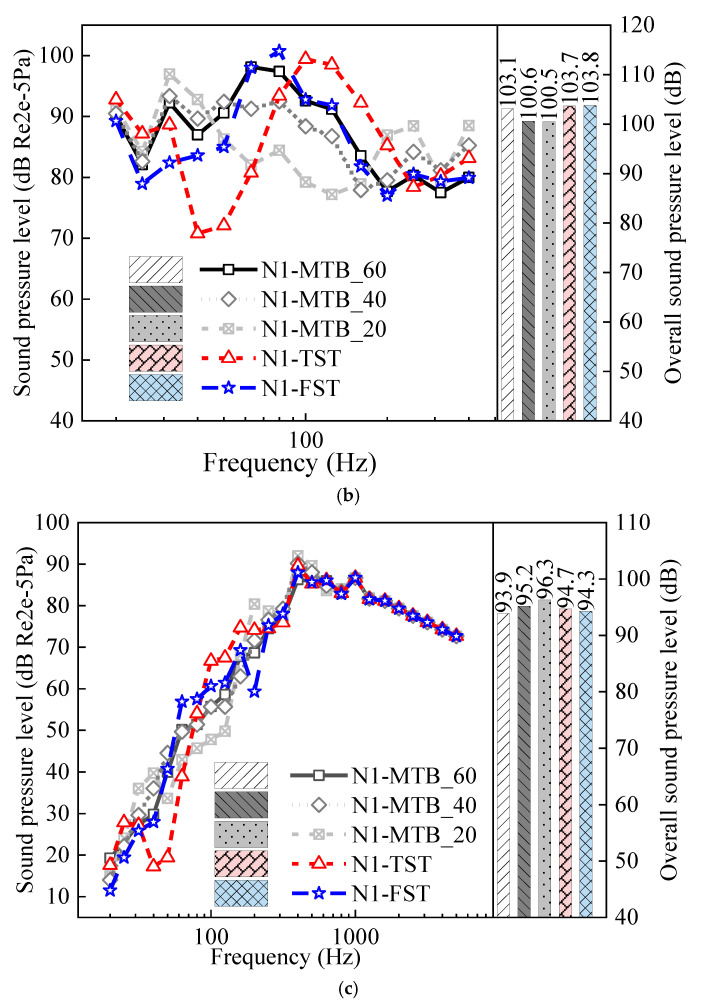
Sound pressure levels for different tracks: (**a**) bridge structure-borne noise; (**b**) track slab noise; (**c**) wheel–rail noise.

Figure 10 illustrates the spectra of noise from the box-girder, track slab, and wheel–rail with the resilient fastener, trapezoidal sleeper, and steel spring floating slab tracks. It can be observed from Figure 10a that the structure-borne noise of the bridge with the MTB is higher than that of the bridge equipped with resilient fasteners, trapezoidal sleepers, or steel spring floating slabs. Specifically, the steel spring floating slab exhibits the most effective noise reduction, followed by the trapezoidal sleeper, reducing the overall sound pressure level of structure-borne noise by 24.6 dB and 8.8 dB, respectively. For every 20 × 10^6^ N/m decrease in the stiffness of the fastener, the total sound pressure level of the bridge structure-borne noise decreases by approximately 2.1 dB. However, the supporting spring force with the trapezoidal sleeper in the frequency band below 31.5 Hz is relatively large, leading to an increase in structure-borne noise within this frequency range. With the reduction in the stiffness of the fastener, the peak force transmitted to the bridge structure decreases. This also leads to a shift in the peak frequency and an increment in the sound pressure level of the bridge structure-borne noise below 50 Hz. In other words, low-stiffness fasteners change the spectrum of the bridge vibration, thereby increasing low-frequency noise.

The sound pressure levels of the track slab noise radiation under the three types of tracks (resilient fasteners, trapezoidal sleepers, and steel spring floating slabs) show distinct variations in their frequency domains. Specifically, the use of resilient fasteners reduced the overall sound pressure level of the track slab noise by approximately 2.5 dB. However, due to significant variations in the peak value of the wheel–rail forces within the low-frequency range, the radiated noise from the trapezoidal sleepers and steel spring floating slabs increased by approximately 0.6 to 0.7 dB.

The alterations in the track structure primarily influenced the wheel–rail noise below 1000 Hz, and the wheel–rail noise above 1000 Hz remained unchanged. Although the modifications to the track structure resulted in substantial variations in the low-frequency wheel–rail noise, the change in the overall sound pressure level was not pronounced because the amplitude of the low-frequency noise was relatively small compared to that in the medium- and high-frequency bands. The sound pressure levels of the wheel–rail noise for the TST and FST were 0.8 dB and 0.4 dB higher than those for MTB_60, respectively. In the case of the MTB, reducing the stiffness of the fasteners resulted in an increment in the overall sound pressure level of the wheel–rail noise for MTB_40 and MTB_20 by 1.3 dB and 2.4 dB, respectively. Despite the increase in wheel–rail noise under resilient tracks, the dominant frequency band of noise adjacent to the track remained 20–315 Hz, which was predominantly influenced by bridge structure-borne noise and track slab noise radiation.

### 4.3. Variations in the Sound Pressure Levels of the Total Noise

**Figure 11:**
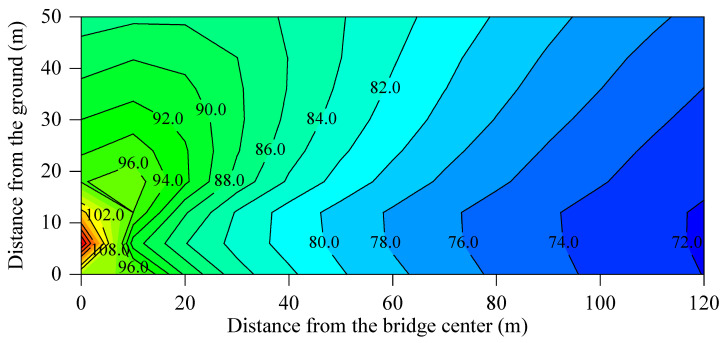
Contour map of the total noise at the mid-span section of the box-girder bridge with MTB_60.

**Figure 12:**
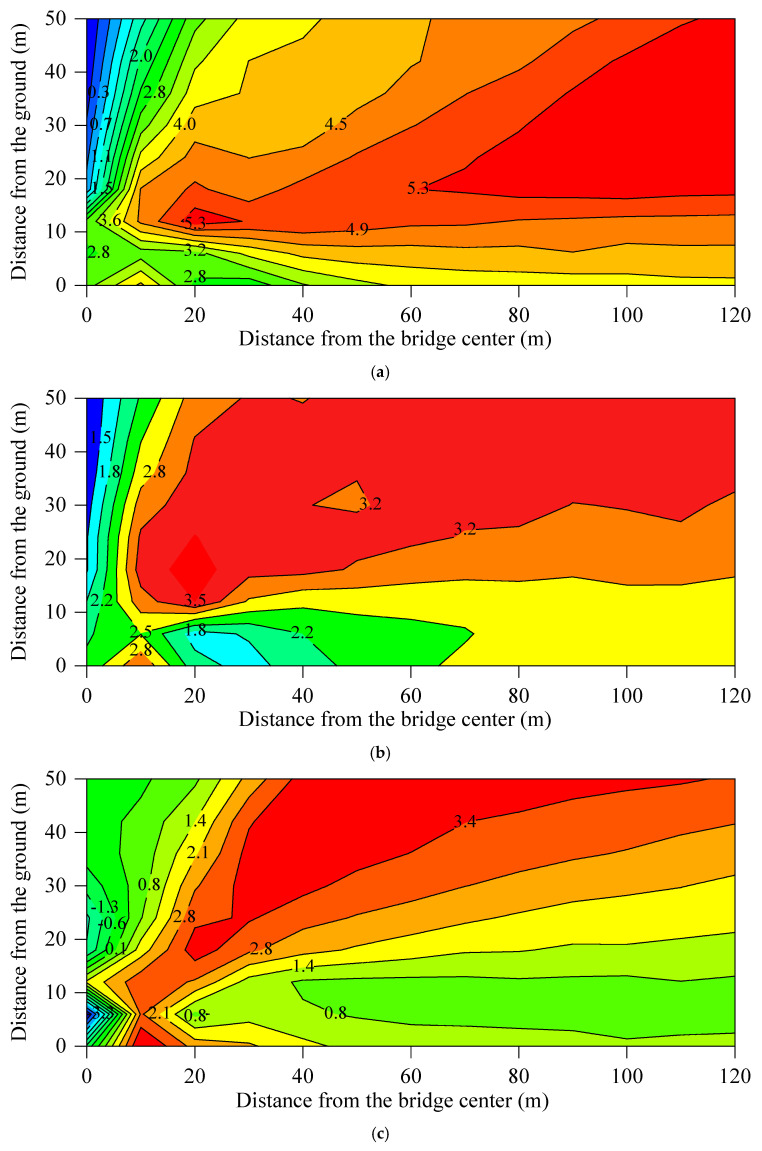

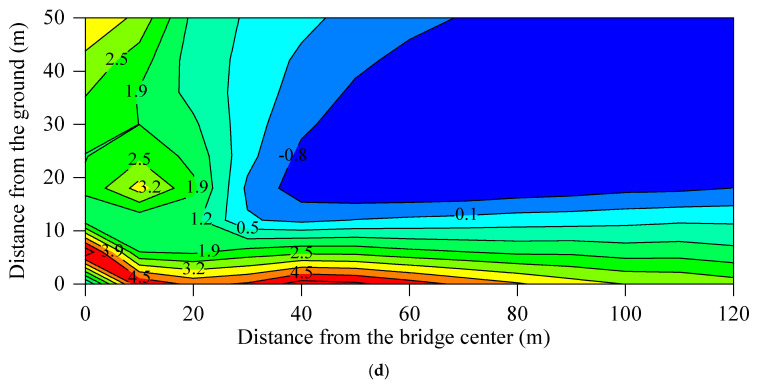
Contour map of the insertion loss at the mid-span section of the box-girder for various tracks: (**a**) MTB_20; (**b**) MTB _40; (**c**) TST; (**d**) FST.

The modifications to the elastic components altered the excitation loads on the bridge structure, track slab, and wheel–rail interface, consequently leading to variations in the spectral characteristics of sound pressure levels across the different track structures. In the actual situation, the sound pressure at a specific spatial location is the cumulative effect of multiple concurrent sound sources. To investigate the propagation patterns of bridge-borne noise, track slab radiation noise, and wheel–rail noise within the sound field, the superimposed sound fields for these sources under various track structures were calculated. Figure 11 illustrates the overall sound pressure level distribution of the total noise at the mid-span section of the bridge with MTB_60. Figure 12 shows the insertion loss distributions at the same section for the MTB_20, MTB_40, TST, and FST, using the overall sound pressure level of the total noise of the viaduct, with MTB_60 as a reference.

For the MTB_60, Figure 10 indicates that bridge-borne noise and track slab noise radiation are the primary sources of elevated urban rail transit noise. Figure 12a,b demonstrates that the bridge structure-borne noise and track slab radiation noise decrease, while the wheel–rail noise increases, as the stiffness of the fasteners decreases. The reduction in bridge structure-borne noise and track slab radiation noise exceeds the increase in wheel–rail noise. Consequently, noise pollution around the viaduct is significantly mitigated. The insertion loss of the total noise at the box-girder side of the MTB with a fastener stiffness of 20 × 10^6^ N/m and 40 × 10^6^ N/m is 1.4–5.7 dB and 1.7–3.3 dB, respectively. However, the reduction in stiffness of the fastener causes the peak frequency of the track slab noise radiation to shift to a lower frequency. In other words, the peak frequency of the noise radiated from the track slab installed with high-stiffness fasteners is higher than that of the track slab with low-stiffness fasteners. In the low-frequency range, the track slab noise will increase to some extent, resulting in an insertion loss above the track slab in Figure 12a that is smaller than that in Figure 12b.

The radiation noise of the track slab for the TST is the main source of the box-girder side noise. Compared to the MTB_60, the insertion loss of the girder side noise for the TST ranges from 0.3 to 4.2 dB. However, the track slab noise from the TST is marginally higher than that of the MTB_60, which is proven by the negative insertion loss observed in Figure 12c.

The radiation noise of the track slab for the FST is greater than that of the MTB_60. The bridge structure-borne noise and the track slab noise of the MTB_60 are approximately the same in magnitude. Therefore, the insertion loss of the total noise of the viaduct with FST in the upper far field is negative, while the insertion loss in the near field and the area below a height of 10 m is positive.

## 5. Conclusions

An assembled model was used to simulate the noise of elevated urban rail transit paved with three types of resilient tracks. The influence of track parameters on bridge structure-borne noise, track slab noise, and wheel–rail noise was discussed. The conclusions are as follows:

(1) The elastic components of the resilient track exert significant influence on the box-girder structure-borne noise. Variations in track configurations alter the amplitude and spectral characteristics of forces transmitted from the wheel–rail interface to the bridge, thereby modifying the distribution of structure-borne noise. The noise reduction effects on bridge structure-borne noise, ranked from largest to smallest, are FST, TST, MTB_20, and MTB_40. As the stiffness of the fastener decreases, the bridge structure-borne noise slightly increases in the frequency band below 50 Hz.
(2) As an effective vibration reduction measure installed beneath the rail, the resilient fastener can mitigate noise radiated from the track slab by approximately 2.5 dB. In contrast, the radiated noise from the track slab of the TST and FST is approximately 0.6 dB higher than that from MTB_60 due to the increasing peak value of wheel–rail forces in the low-frequency range.
(3) The alteration in track structure primarily influences wheel–rail noise below 1000 Hz, whereas it does not significantly change the wheel–rail noise produced above 1000 Hz. Specifically, the application of resilient fasteners results in an increase of 1.3 dB and 2.4 dB in wheel–rail noise. Additionally, the sound pressure levels of the wheel–rail noise for the TST and FST are 0.8 dB and 0.4 dB higher than that of MTB_60.
(4) The structure-borne noise from the box-girder and the track slab are significant contributors to the total noise of viaducts with MTB_60. As the stiffness of the fasteners decreases, the overall sound pressure level of the track slab’s noise radiation increases. This suggests that the stiffness of the fasteners should be optimally matched with other components to achieve the maximum reduction in comprehensive noise.
(5) The slab noise radiation from the TST and FST is higher than the structure-borne noise and wheel–rail noise. The total noise from viaducts paved with FSTs exceeds that of the MTB_60 in the upper far field. This suggests that installing sound barriers on the box-girder is necessary to mitigate radiated noise from the track slab. Noise reduction for elevated urban rail transit requires the integration and coordination of multiple measures to achieve maximum noise mitigation outcomes.

Actual track performance will degrade during service, and time-dependent track parameters can alter track dynamics and noise emission characteristics, which are not involved in this study. To address this issue, future research will focus on incorporating the time-dependent degradation of track properties and conducting extensive field measurements under diverse conditions.

## Figures and Tables

**Table 1:** Calculation parameters of the vehicle–track–bridge interaction model.

Component		Parameter	Unit	Value
Vehicle	Wheel	Young’s modulus	Pa	2.06 × 10^11^
		Diameter	mm	840
		Density	kg/m^3^	7850
		Poisson’s ratio	−	0.3
		Mass of the wheelset	kg	1744
		Unit length mass of the wheel tread	kg/m	63.4
		Thickness of the wheel web	m	0.025
		Wheelbase	mm	2500
	Bogie	Length between the centers of the bogies	m	15.7
		Mass	kg	2430
		Stiffness of the primary suspension	N/m	1.25 × 10^6^
		Damping of the primary suspension	N∙s/m	10,000
Track	Rail	Young’s modulus	Pa	2.06 × 10^11^
		Section moment of inertia	m^4^	3.2 × 10^−5^
		Shear modulus	Pa	7.7 × 10^10^
		Density	kg/m^3^	7850
		Cross area	mm^2^	7745
		Poisson’s ratio	−	0.3
		Loss factor	−	0.01
	Normal fastener	Stiffness	N/m	6 × 10^7^
		Loss factor	−	0.25
		Fastener spacing	m	0.625
	Resilient fastener 1	Stiffness	N/m	2 × 10^7^
	Resilient fastener 2	Stiffness	N/m	4 × 10^7^
	Monolithic track bed	Young’s modulus	Pa	3.45 × 10^10^
		Density	kg/m^3^	2500
		Loss factor	−	0.25
		Length, width, and height	m	3.5 × 1.05 × 0.2
		Stiffness of the supporting spring	N/m	6.89 × 10^10^
		Spacing of the supporting spring	m	1.2
		Loss factor of the supporting spring	−	0.25
	Trapezoidal sleeper track	Young’s modulus	Pa	3.5 × 10^10^
		Density	kg/m^3^	2500
		Loss factor	−	0.02
		Length, width, and height	m	7 × 0.48 × 0.2
		Stiffness of the supporting spring	N/m	2 × 10^7^
		Spacing of the supporting spring	m	1.2
		Loss factor of the supporting spring	−	0.25
	Floating slab track	Young’s modulus	Pa	3.5 × 10^10^
		Density	kg/m^3^	2500
		Loss factor	−	0.02
		Length, width, and height	m	3.5 × 2.1 × 0.3
		Stiffness of the supporting spring	N/m	6 × 10^6^
		Spacing of the supporting spring	m	1.2
		Loss factor of the supporting spring	−	0.25
Bridge	Main girder	Length	m	30
		Height	m	1.7
		Width of the deck	m	9.3
		Width of the bottom	m	4
		Young’s modulus	Pa	3.45 × 10^10^
		Density	kg/m^3^	2500
		Loss factor	−	0.02
		Poisson’s ratio	−	0.2

## Data Availability

The data presented in this study are available upon request from the corresponding author due to privacy and adherence to collaboration agreements.

## References

[1] Liu Q.M., Gao K., Song L.Z., Liu L.Y., Luo Y.K. (2024). Investigation on multiple traffic noise near an airport and their effect on nearby residents. Sci. Rep..

[2] Thompson D.J. (1993). Wheel-rail noise generation Ⅰ: Introduction and interaction model. J. Sound Vib..

[3] Song L.Z., Gao K., Liu Q.M., Zhang L.T., Feng Q.S., Guo W.J. (2022). Acoustic performance of near-rail low-height noise barriers installed on suburban railway bridges. Environ. Sci. Pollut. R..

[4] Saurenman H., Phillips J. (2005). In-service tests of the effectiveness of vibration control measures on the BART rail transit system. J. Sound Vib..

[5] Gao X.G., Yan H.D., Feng Q.S., Ma Y.F., Du M.J. (2025). Performance research and effect evaluation of a high-grade damping fastener in subway line. Eng. Fail. Anal..

[6] Song X.D., Li Q. (2018). Numerical and experimental study on noise reduction of concrete LRT bridges. Sci. Total Environ..

[7] Sadeghi J., Seyedkazemi M., Khajehdezfuly A. (2020). Nonlinear simulation of vertical behavior of railway fastening system. Eng. Struct..

[8] Li X.Z., Liang L., Wang D.X. (2018). Vibration and noise characteristics of an elevated box girder paved with different track structures. J. Sound Vib..

[9] Yan B., Pan L., Xu L., Deng X.Y., Liu W., Du X.G. (2023). Dynamic performance analysis of floating slab track system considering flexible wheelset. Constr. Build. Mater..

[10] Liang L., Li X.Z., Yin J., Wang D.X., Gao W., Guo Z. (2019). Vibration characteristics of damping pad floating slab on the long-span steel truss cable-stayed bridge in urban rail transit. Eng. Struct..

[11] Liang L., Li X.Z., Zheng J., Lei K.N., Gou H.Y. (2020). Structure-borne noise from long-span steel truss cable-stayed bridge under damping pad floating slab: Experimental and numerical analysis. Appl. Acoust..

[12] Liu X.Z., Wang Y., Yin Z.R., Gao T.C., Luo Q. (2024). In-situ measurement of subway train-induced vibration and noise of steel spring floating slab with MEMS-based sensing units. Meas. Sci. Technol..

[13] Zhao Z.M., Wei K., Ding W.H., Du W., Li H.L. (2021). UM-SIMULINK Co-simulation for the vibration reduction optimization of a magnetorheological damping steel-spring floating slab track. Constr. Build. Mater..

[14] Huang X.D., Zeng Z.P., Wang D., Luo X.W., Li P., Wang W.D. (2023). Experimental study on the vibration reduction characteristics of the floating slab track for 160 km/h urban rail transit. Structures.

[15] He W., Zou C., Pang Y.T., Wang X.M. (2020). Environmental noise and vibration characteristics of rubber-spring floating slab track. Environ. Sci. Pollut. R..

[16] Song L.Z., Gao K., Liu Q.M., Liu L.Y., Feng Q.S. (2023). Study on the structure-borne noise of U-shaped girder bridges with fully-enclosed sound barriers. Appl. Acoust..

[17] Li X.Z., Hu X.H., Zheng J. (2020). Statistical energy method for noise reduction performance of the vertical noise barrier alongside railway bridges. Appl. Acoust..

[18] Zhang Y.F., Li L., Lei Z.Y., Yu L.B., Bu Z. (2021). Environmental noise beside an elevated box girder bridge for urban rail transit. J. Zhejiang Univ. Sci. A..

[19] Thompson D.J. (2009). Railway Noise and Vibration: Mechanisms, Modelling and Means of Control.

[20] Li X.Z., Zhang X., Zhang Z.J., Liu Q.M., Li Y.D. (2015). Experimental research on noise emanating from concrete box-girder bridges on intercity railway lines. Proc. Inst. Mech. Eng. F J. Rail Rapid Transit.

[21] Zhang X., Li X.Z., Song L.Z., Su B., Li Y.D. (2016). Vibrational and acoustical performance of concrete box-section bridges subjected to train wheel-rail excitation: Field test and numerical analysis. Noise Control Eng. J..

[22] Song L.Z., Li X.Z., Hao H., Zhang X. (2018). Medium- and high-frequency vibration characteristics of a box-girder by the waveguide finite element method. Int. J. Struct. Stab. Dyn..

[23] Thompson D.J. (1993). Wheel-rail noise generation, Part V: Inclusion of wheel rotation. J. Sound Vib..

[24] Thompson D.J., Hemsworth B., Vincent N. (1996). Experimental validation of the TWINS prediction program for rolling noise, Part Ⅰ: Description of the model and method. J. Sound Vib..

[25] Zhang X.Y., Squicciarini G., Thompson D.J. (2016). Sound radiation of a railway rail inclose proximity to the ground. J. Sound Vib..

[26] Zhang X.Y., Thompson D.J., Squicciarini G. (2016). Sound radiation from railway sleepers. J. Sound Vib..

[27] Zhang X.Y., Thompson D.J., Jeong H., Squicciarini G. (2017). The effects of ballast on the sound radiation from railway track. J. Sound Vib..

[28] Zhang X.Y., Jeong H., Thompson D.J., Squicciarini G. (2019). The noise radiated by ballasted and slab tracks. Appl. Acoust..

[29] Zhang X.Y., Thompson D.J., Ryue J., Jeong H., Squicciarini G. (2023). The effect of rail shields on railway rolling noise. Int. J. Rail Transp..

[30] Thompson D.J., Jones C.J.C. (2000). A review of the modelling of wheel/rail noise generation. J. Sound Vib..

[31] He Y.P., Zhou Q., Xu F., Sheng X.Z., He Y.L., Han J. (2023). An investigation into the effect of rubber design parameters of a resilient wheel on wheel-rail noise. Appl. Acoust..

[32] Song L.Z., Zhang J., Liu Q.M., Zhang L.T., Wu X.L. (2024). Characteristics of noise caused by trains passing on urban rail transit viaducts. Sustainability.

[33] Brebbia A.C. (2017). The birth of the boundary element method from conception to application. Eng. Anal. Bound. Elem..

[34] Zhang X.A., Zhai W.M., Chen Z.W., Yang J.J. (2018). Characteristic and mechanism of structural acoustic radiation for box girder bridge in urban rail transit. Sci. Total Environ..

[35] Lyon R.H. (1975). Statistical Energy Analysis of Dynamical Systems: Theory and Applications.

[36] Liu Q.M., Thompson D.J., Xu P.P., Feng Q.S., Li X.Z. (2020). Investigation of train-induced vibration and noise from a steel-concrete composite railway bridge using a hybrid finite element-statistical energy analysis method. J. Sound Vib..

[37] Liu Q.M., Liu L.Y., Chen H.P., Zhou Y.L., Lei X.Y. (2020). Prediction of vibration and noise from steel/composite bridges based on receptance and statistical energy analysis. Steel Compos. Struct..

[38] Harrison M.F., Thompson D.J., Jones C.J.C. (2000). The calculation of noise from railway viaducts and bridges. Proc. Inst. Mech. Eng. F J. Rail Rapid Transit.

[39] Sheng X.Z., Cheng G., Thompson D.J. (2020). Modelling wheel/rail rolling noise for a high-speed train running along an infinitely long periodic slab track. J. Acoust. Soc. Am..

[40] Yang X.W., Shi G.T. (2014). The effect of slab track on wheel/rail rolling noise in high speed railway. Intell. Autom. Soft Comput..

[41] Zhou Q., He Y.P., Li M.X., Liu Z., He Y.L., Sheng X.Z. (2022). A parametric study on the structural noise radiation characteristics of a steel spring floating slab track. Adv. Mech. Eng..

[42] (2013). Acoustic–Railway Applications–Measurement of Noise Emitted by Railbound Vehicles.

